# Introduction to *Cannabis and Cannabinoid Research*

**DOI:** 10.1089/can.2015.29002.edi

**Published:** 2016-01-01

**Authors:** Daniele Piomelli

**Affiliations:** ^1^ Department of Anatomy and Neurobiology, University of California, Irvine, Irvine, California.; ^2^ Department of Drug Discovery and Development, Istituto Italiano di Tecnologia, Genova, Italy.

It is with great pleasure that I welcome you to the first issue of *Cannabis and Cannabinoid Research*, a peer-reviewed journal entirely dedicated to the scientific, medical, and psychosocial exploration of clinical Cannabis, cannabinoids, and the endocannabinoid system.

We have known about the psychoactive properties of Cannabis for thousands of years. A rich archaeological and literary record bears witnesses to its utilization by human societies throughout the Asian continent since at least the third millennium BCE. Like other psychoactive plants, in antiquity, Cannabis was used primarily as a medicine, in the distinctively religious sense premodern cultures gave to this word: its use was a prerogative of the shaman and the physician-priest rather than the common person.

Many psychoactive remedies of the ancient world have found their way into modern medical science—think about *Papaver somniferum* (opium), *Atropa belladonna* (deadly nightshade), and *Erythroxylum coca* (coca), just to cite a few. However, not Cannabis. Largely ignored by Greco–Roman physicians and their Renaissance followers, the plant made a timid reappearance in European pharmacopeias of the XIX and XX centuries, only to be excluded again after a few decades. The societal pressures that caused the demise of Cannabis as a medicine are unclear—and deserving of serious study—as are the forces that underlie its progressive resurfacing in the last two decades. Today, the public opinion in many countries of the Western hemisphere is undergoing a radical shift from viewing Cannabis as a dangerous street drug to considering it a relatively safe medicine—or even a natural cure-all.

Medical science is not fully prepared for this sea change. To be sure, in the last 50 years, basic and clinical researchers have fought intermittent funding and irrational Federal regulations to unravel the actions of Cannabis on the human body, its medical benefits, and the risks it poses to users. This hard work has paid out. We have now a reasonably complete catalogue of the chemicals present in the plant; we have discovered receptors through which one of its main components, Δ^9^-tetrahydrocannabinol (THC), changes cell functions; and we have unveiled the existence of a previously unknown neurotransmitter system that underpins the actions of THC and we are learning ways to leverage this system to create new medicines. We have also been able to accumulate a small body of hard facts about the impact—both positive and negative—of Cannabis on human health. However, many questions remain unanswered, and there is still much more to be done.

Now, more than ever, we need hard scientific facts to guide our societal choices about Cannabis. And, as always, the only way to separate facts from opinions is to combine sound experiments with unfettered critical discussion. *Cannabis and Cannabinoid Research* was created to help achieve this goal. Its objective is to diffuse high-quality basic and clinical research and scholarly debates on Cannabis, its chemical constituents, and the physiological systems these substances impact upon.

Cannabinoid researchers have of course many other means to publish their work—there is no dearth of outstanding journals in basic and clinical neuroscience and pharmacology. We do not aspire to replace them. What we intend to do, however, is to open a space where critical discussions—in the form of round tables, commentaries, and interviews—and informative research studies touching all aspects of cannabinoid research can find their place. Our approach will be interdisciplinary—spanning from chemical to social sciences—as demanded by the complex history of the human interaction with Cannabis, the varied effects exerted by this plant on the human body, and the pervasive roles played by the endocannabinoid system in physiology and pathology.

I feel honored to have been asked to serve as the editor in chief of *Cannabis and Cannabinoid Research*, and I am extremely pleased to welcome Drs. Allyn Howlett, Mauro Maccarrone, Margaret Haney, and Markus Leweke as Associate Editors. We are fortunate that so many other leaders in the cannabinoid field have agreed to serve in the journal's editorial board: their scientific standing and broad expertise capture in a unique way our aspirations for rigor and interdisciplinary breadth. Finally, my thanks go to the publisher, Mary Ann Liebert, Inc., whose vision has made this venture possible.

## Abbreviations Used

BCE: before the common era
THC: Δ^9^-tetrahydrocannabinol

